# RACIAL/ETHNIC DIFFERENCES IN SUBJECTIVE COGNITIVE DECLINE SYNDROMES

**DOI:** 10.1093/geroni/igae098.3324

**Published:** 2024-12-31

**Authors:** Moyosoreoluwa Jacobs, Peter Lichtenberg, Wassim Tarraf

**Affiliations:** Wayne State University, Melvindale, Michigan, United States; Wayne State University, Detroit, Michigan, United States; Wayne State University, Detroit, Michigan, United States

## Abstract

Subjective cognitive decline (SCD) is a precursor to Alzheimer’s Disease and Related Dementias (ADRD). SCD rates vary by race/ethnicity. SCD is also heterogenous in the population and can yield different syndromes. We used Behavioral Risk Factor Surveillance System data (2015/2016 and 2019/2020) from non-Hispanic White (NHW), Black (NHB), and Hispanic individuals aged 50+ years (unweighted-n=44,152) who endorsed SCD, to fit latent class analysis (LCA) and derive SCD syndromes based on measures of activities limitations, need for assistance, and consulting with healthcare providers. We used multinomial logistic regression to model differences in syndrome prevalence by R/E and assess the contributions of predisposing (e.g., age), enabling (e.g., insurance), and health need (E.g., cardiovascular) factors to explaining these racial/ethnic (R/E) differences. Three groupings (social limitations [13.3%], mixed functional limitations [26.2%] and uncomplicated [60.5%]) fit the data well. Compared to NHWs (21.9%), Hispanics and NHBs were significantly more likely to meet criteria for mixed functional limitations [38.2% and 41.2% respectively] and less likely to be in the uncomplicated group. Predisposing, enabling, and health need factors only partially attenuated (10-22%) the Relative Risk Ratios for classification in the mixed functional limitations for NHBs and Hispanics. Hispanics and NHBs presented with more mixed SCD-related functional limitations compared to NHWs, suggesting higher risk for developing ADRDs. Since nearly 2/3 individuals in the mixed functional limitations group reported discussing SCD with healthcare providers, there are opportunities to provide interventions for this high risk group at their place of care, and reduce R/E disparities in SCD and ADRD.

